# HPV positive tonsillar cancer in two laser surgeons: case reports

**DOI:** 10.1186/1916-0216-42-54

**Published:** 2013-11-18

**Authors:** Margo Rioux, Andrea Garland, Duncan Webster, Edward Reardon

**Affiliations:** 1 Dalhousie Medicine New-Brunswick, 100 Tucker Park Road, Saint John, New-Brunswick E2L 4 L5, Canada; 2 Saint John Regional Hospital, PO Box 2100, Saint John, New-Brunswick E2L 4 L2, Canada

**Keywords:** Laser plume, Human Papillomavirus, Squamous cell carcinoma, Tonsillar cancer, Oropharyngeal cancer

## Abstract

A 53 year-old male gynecologist presented with human papillomavirus (HPV) 16 positive tonsillar squamous cell carcinoma. He had no identifiable risk factors with the exception of long term occupational exposure to laser plumes, having performed laser ablations and loop electrosurgical excision procedures (LEEP) on greater than 3000 dysplastic cervical and vulvar lesions over 20 years of practice. The second patient is a 62 year old male gynecologist with a 30 year history of laser ablation and LEEP who subsequently developed HPV 16 positive base of tongue cancer. He also had very few other risk factors for oropharyngeal cancer or HPV infection. HPV is a probable causative agent for oropharyngeal squamous cell carcinoma and has been reported as being transmittable through laser plume. This paper suggests that HPV transmitted through laser plume can result in subsequent squamous cell carcinoma.

## Background

Carbon dioxide (CO2) lasers are commonly used to excise lesions on the larynx, cervix, lower genital tract, and perianal regions. Although this is an effective treatment modality, there are concerns regarding potential adverse events associated with its use. Tissue destruction from the laser's energy produces a gaseous plume containing cell contents and other aerosols. Many potential risks have been associated with laser plume exposure including the risk of human (HPV) transmission; *in vitro* experiments have reported HPV transmission through laser plumes. Furthermore, there are two case reports describing health care professionals contracting oropharyngeal HPV following long-term occupational exposure to laser plumes. Certain HPV subtypes are known to be oncogenic, therefore, laser plume exposure may put health care professionals at increased risk of oropharyngeal carcinoma development. This case report describes, to our knowledge, the first cases of HPV-16 positive oropharyngeal squamous cell carcinomas in two surgeons following long-term occupational laser plume exposure.

## Case presentations

Patient A is a 53 year-old male gynaecologist who consulted the Department of Otolaryngology having noticed a lesion on his right tonsil and a lump in the right side of his neck. His symptoms had started a few months prior to his visit and included increased fatigue. The physical exam revealed right tonsil hypertrophy and a small node at the posterior aspect of the right mandible. A CT scan of the region demonstrated a 2.2 cm soft tissue lesion in the right tonsil extending to the right soft palate; it also confirmed the presence of a level 2 lymph node. A biopsy of the right tonsil was performed confirming invasive squamous cell carcinoma of moderate to poor differentiation. The cancer was staged at T2N1M0. Testing at the National Microbiology Laboratory in Winnipeg, Manitoba revealed that the lesion was positive for HPV type 16 by hybrid capture assay (Luminex Hybridization).

Patient A had no identifiable risk factors for oropharyngeal cancer or HPV with the exception of occupational exposure to HPV-positive laser plumes, having performed laser ablation and later loop electrosurgical excision procedures (LEEP) of more than 3000 dysplastic cervical and vulvar lesions over 20 years. Most of these procedures were performed in an environment without proper ventilation or mask. Being in a long-term monogamous relationship with his wife, he denies any other sexual contacts. She was tested following patient A’s diagnosis and was found to be HPV negative. Unfortunately, she had no HPV testing prior to patient A’s cancer diagnosis. Additionally, the patient is a non-smoker and consumes alcohol only occasionally. He has never been vaccinated against HPV. The association of his diagnosis with workplace exposure warranted his receipt of worker's compensation.

He received Intensity Modulated Radiotherapy over 35 fractions with adjuvant cisplatin for 3 courses. Treatment side-effects caused significant reduction in quality of life due to marked dysphagia and xerostomia, which resulted in 40 lbs weight loss despite percutaneous gastrostomy feeding, radiation-induced Lhermitte's sign, and 40% high frequency hearing loss. He returned to work 11 months following initiation of therapy. In the four years since treatment, he has had no recurrence of squamous cell carcinoma; he was subsequently vaccinated against HPV types 6, 11, 16 and 18. He continues to practice gynecology, but has not returned to laser surgery.

Patient B is a 62 year old gynecologist who recently consulted his local otolaryngologist after having a foreign body sensation in his throat for many weeks. A biopsy of the base of tongue revealed a squamous cell carcinoma; the lesion was positive for HPV 16. The base of tongue lesion was excised by laser and he also had a bilateral modified neck dissection. He had been practicing for 30 years, of which he spent 15 doing weekly laser ablations with a CO2 laser. He reported poor ventilation in this clinic space and subsequently moved to a different area where he performed loop electrosurgical excision procedures for the next 15 years. He is a non-smoker, only drinks occasionally, and was married twice. Once again, this patient may have contracted HPV through occupational laser plume exposure.

## Discussion

Squamous cell carcinomas are accountable for 90% of head and neck cancers [1]. Tobacco smoke, alcohol, and HPV exposure are the strongest risk factors associated with head and neck squamous cell carcinomas; however, family history, low socioeconomic status, Epstein-Barr virus, acquired immunodeficiency syndrome (AIDS), and human immunodeficiency virus (HIV) status are also minor risk factors [1]. Review of Patient A's history reveals that he had no known risk factors for head and neck squamous cell carcinoma other than being HPV-positive. Although it is impossible to confirm, the patient denies other sexual contact. He is in a long term monogamous with his wife and swabs collected from patient A’s wife following his cancer diagnosis where negative for HPV. In the absence of other risk factors, it is possible that the source of HPV infection could be due to occupational laser plume exposure. Patient B was also a non-smoker with only occasional alcohol consumption. He was, however, married twice and the HPV status of these partners was not established. There is an abundance of evidence in the literature supporting HPV's role in the oncogenesis of head and neck squamous cell carcinomas, as well as strong evidence supporting HPV transmission through laser plume.

The potential for HPV transmission through laser plume has been explored by various authors. Many studies have reported the presence of intact HPV DNA in laser plume of HPV-positive lesions; however, other studies have not been able to recreate these findings [2]. Two case reports describe HPV infection in two health care professionals regularly exposed to laser plumes in an occupational setting. Both reported no other risk factors or other possible exposures to the virus [3,4]. Furthermore, there is a significantly increased incidence of nasopharyngeal warts in laser surgeons when compared to a control group [5]. HPV transmission and subsequent tumour growth has been demonstrated in bovines inoculated with laser plume produced by destruction of HPV-positive tissue [6]. This presents a strong argument in favour of HPV transmission through laser plume.

The relationship between HPV and oropharyngeal squamous cell carcinomas has been studied extensively. A study of the American SEER database from 1984 to 2004 reveals that HPV-positive head and neck cancers have increased 225% during the study period, while HPV-negative head and neck cancers have decreased by 50% [7]. Retrospective studies in Canada [8] and Australia [9] both report an increase of cancer in sites potentially related to HPV infection, such as the base of the tongue and the tonsils, when compared to sites not associated with HPV. A Canadian study has reported that 73% of cancers of the base of the tongue and tonsils were HPV-positive [10]. Oral Squamous cell carcinomas are strongly associated with HPV 16 and HPV 18 [11]. Reviews have concluded that existing evidence points towards a clinically significant [12] and causal relationship [13] between HPV and oropharyngeal squamous cell carcinoma.

Patient A's lesion was a poor to moderately differentiated cancer with a stage of T2N1M0. His cancer responded well to treatment and he has not had any relapse. HPV-positive and HPV-negative oropharyngeal squamous cell carcinomas seem to be two distinct types of cancer as they have different histological appearances [14] and genetic signatures [15]. HPV-positive tumours tend to present at a higher stage, [7] however, they seem to be associated with a higher survival rate [7,16,17], and a better response to treatment [18]. Despite this increased survival rate and response to treatment, it is not recommended to modify the management of HPV-positive oropharyngeal squamous cell carcinoma as there is not enough high quality evidence to support it at this time [19,20].

## Conclusions

This article reports on a case of HPV-16 positive oropharyngeal squamous cell carcinoma in two laser surgeons following occupational exposures to laser plumes. There is now a strong body of evidence supporting a causal relationship between oncogenic HPV types and head and neck squamous cell carcinomas. It is also recognized that HPV may be transmitted through laser plume. Therefore, long term occupational exposure to laser plumes may lead to HPV infection and oropharyngeal squamous cell carcinomas. There is significant morbidity associated with these lesions and their treatment. It would thus seem prudent to reduce laser plume exposure amongst healthcare professionals. Existing protective methods such as standard surgical mask and laser mask have been described as ineffective against viral pathogens [21]. The National Institute for Occupational Safety and Health (NIOSH) recommends the use of local exhaust ventilation (LEV) in addition to general room ventilation. Portable smoke evacuators should be used to reduce surgical smoke levels. The device should be fitted with High Efficiency Particulate Air (HEPA) filter or its equivalent, which should be monitored and replaced on a regular basis. The nozzle inlet should be kept within 2 inches of the surgical site and be kept ON at all times when the laser is in use [22]. Furthermore, the Canadian Center for Occupational Health and Safety (CCOHS) recommends the use of respirators (N95 grade or higher) to protect healthcare workers [23]. A 2008 survey of various North American medical facilities indicated that less than half of respondents used effective LEV and even fewer used proper respiratory protective equipment [24]. Therefore, many health care workers are being exposed to oncogenic HPV strains. In the absence of effective screening methods, those infected risk developing oropharyngeal cancer [25]. This highlights the potential role of primary prevention through prophylactic HPV vaccination. Those exposed to laser plume in an occupational setting may benefit from vaccination against oncogenic HPV strains, in order to prevent infection and reduce the risk of subsequent oropharyngeal cancer.

## Consent

Written informed consent was obtained from the patients for publication of the Case report. Copies of written consents are available for review by the Editor-in-chief of this journal.

## Competing interests

The authors declare that they have no competing interests.

## Authors’ contributions

MR has reviewed the patient's chart as well as the literature and drafted the manuscript. AG has conceived this case report, participated in its coordination and has helped draft the manuscript. DW has helped review the manuscript and provided substantial intellectual content to the original manuscript. ER has helped draft the manuscript. All authors have read and approved the final manuscript.
